# Effects of different seasons on the outcome of *in vitro* fertilization and embryo transfer: a retrospective cohort study

**DOI:** 10.3389/frph.2026.1782049

**Published:** 2026-04-07

**Authors:** Run Zhao, Linlin Che, Qianyan Song, Xiaofang Li, Xiao Li

**Affiliations:** Department of Reproductive Medicine, Affiliated Hospital of Southwest Medical University, Luzhou, Sichuan, China

**Keywords:** antagonist ovulation induction protocol, different seasons, embryo quality, pregnancy outcome, stratification analysis

## Abstract

**Objective:**

We aimed to investigate the effect of seasons on embryo quality and pregnancy outcomes among infertile patients undergoing *in vitro* fertilization-embryo transfer (IVF-ET)-assisted pregnancy.

**Methods:**

We retrospectively analyzed data from 1,413 infertile patients who received IVF and antagonist ovulation induction program in the Reproductive Medicine Center of our hospital from January 2019 to December 2023. Based on seasons, they were divided into the spring group, summer group, autumn group, and winter group. The general information, embryo status, and pregnancy outcome of patients in different groups were compared.

**Results:**

There were no significant differences in general data, embryo quality, and pregnancy outcomes among the four groups (*P* > 0.05). In multivariate binary logistic regression analysis, season did not significantly affect the positive rate of human chorionic gonadotropin (HCG), clinical pregnancy rate, miscarriage rate, and live birth rate. After adjusting beta regression analysis for confounding factors, the blastocyst formation rate in spring was significantly higher than that in summer, the MII oocyte rate in 2019 was significantly higher than that in 2020 and 2022, and the available embryo rate in 2019 was significantly higher than that in 2022 and 2023 (*P* < 0.05). Stratified analysis by year showed that in 2020, the blastocyst formation rate in the spring group and autumn group was significantly higher than that in the winter group. In 2022, the HCG positive rate was significantly higher in the spring group than in the summer and autumn groups, and the clinical pregnancy rate was significantly higher in the spring and winter groups than in the summer group. In 2023, the blastocyst formation rate was significantly higher in the spring and winter groups than in the autumn group (*P* < 0.05).

**Conclusion:**

For infertile patients undergoing controlled ovarian stimulation with an antagonist protocol, the rate of blastocyst formation in the spring was higher than that in the summer. There might be a potential interaction between the year and the season.

## 1 Introduction

Infertility is a major driver of health care burden worldwide. The World Health Organization estimates that there are as many as 48 million couples and 186 million people suffering from infertility (1). The “Survey Report on the Status of Infertility in China” published at the China International Infertility Summit Forum indicated that the infertility rate in China has increased from 2.5% to 3% 20 years ago to 12.5% to 15% in recent years (2). The development of human-assisted reproductive technology (ART) helps many infertile couples to have offspring. It has been confirmed that human fertility and birth rates exhibit seasonal variations (3). However, there are still a few studies on the relationship between seasonal changes and the outcome of IVF-ET-assisted pregnancy. It is not yet clear whether different seasons affect the outcomes of IVF-ET-assisted pregnancy (4, 5). Chamoun et al. (6) found that the pregnancy rate was the lowest in spring in the IVF cycle, while Weigert et al. (7) found that the pregnancy rate was the lowest in July and the highest in December in the IVF cycle. Rojansky et al. (8) showed that the fertilization rate and excellent embryo rate were the highest in spring and the lowest in autumn in the IVF cycle. Braga et al. (9) reported the highest fertilization rate in spring in ICSI cycles, whereas Revelli et al. (10), Wunder et al. (11) and Tomic et al. (12) found no significant seasonal differences in the outcomes of human-assisted reproductive therapy. Perhaps differences in the characteristics of the included subjects, variations in control ovarian stimulation protocols, and differences in the seasonal groupings led to inconsistent results. This study aimed to compare the embryo quality and pregnancy outcomes of infertile patients undergoing controlled ovarian stimulation with an antagonist protocol and explore the effects of different seasons on embryo quality and pregnancy outcomes, thus providing a reference for clinical work.

## 2 Materials and methods

### 2.1 Participants

This study was conducted in April 2025. In total, 1,413 patients undergoing IVF and using the antagonist ovulation promotion program in the Reproductive Medicine Center of our hospital (It is located in the southwest of China and has a subtropical monsoon climate) from January 2019 to December 2023 were enrolled in this study. Based on the national standard “GB/T 42074-2022 Climate Season Classification”, the core defining indicator was the 5-day moving average of daily average temperature. Spring: The first day when the first consecutive 5 days of the 5-day moving average temperature were ≥10 ℃ and <22 ℃, until the day before the threshold switch (5-day moving average temperature ≥22 ℃). Summer: The first day when the first consecutive 5 days of the 5-day moving average temperature was ≥22 ℃, until the day before the threshold dropped (5-day moving average temperature <22 ℃); Autumn: The period when the 5-day moving average temperature was ≥10 ℃ and <22℃ (from the day after the threshold dropped in summer to the day before the threshold switch in winter). Winter: The period when the 5-day moving average temperature was <10 ℃ (from the day after the threshold switch in autumn to the day before the threshold reaches the standard in the following spring). Combined with the daily observation data of the Meteorological Bureau of Luzhou City, Sichuan Province, from 2019 to 2023 (climate statistics followed the unified standard of WMO), the division of the four seasons and climate characteristics from 2019 to 2023 are summarized in Table 1. According to different seasons, the patients were divided into spring group, summer group, autumn group, and winter group. The inclusion criteria were as follows: (1) infertile patients whose days of entering the ovulation induction cycle and the transplantation procedure fell within the same season; (2) antagonist ovulation induction protocol; (3) complete clinical data; (4) Only for patients with tubal infertility; (5) age ≤40 years old; (6) no serious organic lesions; and (7) no immune diseases; (8) The male's semen test was normal. The exclusion criteria were as follows: (1) ovarian dysfunction; (2) repeated implantation failure (The definition of Repeated Implantation Failure (RIF) is based on the “Chinese Expert Consensus on Clinical Diagnosis and Treatment of Repeated Implantation Failure (2023)”: Adult women under the age of 40 who, after three fresh or frozen cycles of transplanting at least three high-quality embryos, still fail to achieve clinical pregnancy); (3) endometriosis, adenomyosis, uterine fibroids; (4) endometrial polyps, uterine adhesions; (5) uterine malformation; (6) recurrent abortion; (7) chromosome abnormality in either party. This study was a retrospective study, and informed consent was waived by the Ethics Committee of the Affiliated Hospital of Southwest Medical University. Ethical approval number: KY2024292.

**Table 1:** Start dates of each season and average relative humidity from 2019 to 2023.

Season	Spring			Summer			Autumn			Winter		
Year	Start and end dates	average temperature	average relative humidity	Start and end dates	average temperature	average relative humidity	Start and end dates	average temperature	average relative humidity	Start and end dates	average temperature	average relative humidity
2019	3.13–5.24	17.5	74%	5.25–9.27	27.2	81%	9.28–11.22	18.2	69%	11.23–2,020.3.12	9.8	64%
2020	3.13–5.25	17.7	75%	5.26–9.28	27.5	80%	9.29–11.22	18.3	68%	11.23–2,021.3.9	10.0	65%
2021	3.10–5.23	17.9	73%	5.24–9.30	27.8	80%	10.1–11.20	18.4	70%	11.21–2,022.3.7	10.1	66%
2022	3.8–5.26	18.0	76%	5.27–10.2	27.7	82%	10.3–11.25	18.6	68%	11.26–2,023.3.7	10.3	65%
2023	3.8–5.24	17.6	74%	5.25–9.29	27.4	79%	9.30–11.23	18.5	67%	11.24–2,024.3.10	10.2	64%

### 2.2 Research methods

#### 2.2.1 Antagonist ovulation induction protocol

Ovulation induction began on days 2–3 of menstruation. Patient's anti-mullerian hormone (AMH), antral follicle count (AFC), age, body mass index (BMI), and previous ovarian response were used to determine gonadotropin (Gn) initiation dose [follicle-stimulating hormone (FSH): 100–300 IU] (13, 14). Antagonist fixed regimen: Antagonist addition began on day 5 or 6 of Gn use, but varied depending on the specific characteristics of the patients. Antagonist flexibility: The timing of antagonist initiation was determined based primarily on follicle size and hormone levels, either when the dominant follicle diameter was 14 mm or 15 mm, or when the dominant follicle diameter was >12 mm and serum estradiol level was >300 ng/L (1 ng/L = 3.672 pmol/L) and continued until the trigger day. Triggers were introduced when 3 dominant follicles were ≥17 mm in diameter or 2 dominant follicles were ≥18 mm in diameter, and eggs were retrieved 35–37 h after the trigger (15). Luteal support started on the day of retrieval (16) [oral dydrogesterone tablets (Dufton, Abbott, Netherlands) 10 mg tid, intramuscular progesterone injection (Zhejiang Xianju, China) 60 mg qd, or oral dydrogesterone tablets 10 mg tid and vaginal progesterone sustained-release gel (Schnaughton, Merck Serono Co., Ltd., Germany) 90 mg qd). The decision for fresh embryo transfer was made based on the patient's wishes, the condition of the embryo, endometrial condition, and the level of sex hormones. Cleavage embryo transfer was conducted on the third day after oocyte retrieval, blastocyst transfer was performed on the fifth day after oocyte retrieval, and luteal support was continued after embryo transfer.

#### 2.2.2 Embryo culture and transfer

IVF/ICSI insemination and fertilization were observed 16–18 h after insemination. Cleavage was observed on the second and third days. The embryo scoring standard at the cleavage stage referred to assisted reproduction laboratory technology edited by Huang Guoning. High-quality embryos refer to those derived from 2PN that have developed into 7–9 cells on the third day and have a morphology of grade I or grade II. If the culture was continued to the 5th or 6th day and developed to the blastocyst stage, the blastocyst was scored based on the blastocyst scoring standard (17). The blastocyst scoring system mainly adopted the Gardner scoring system (18), and the high-quality embryos were blastocysts with grade 3BB and above. The valid embryos were transferred according to the patients' age and IVF indications. The remaining embryos were frozen and stored.

#### 2.2.3 Measurement of endometrial thickness during transplantation procedure

In this study, the measurement of endometrial thickness during transplantation strictly followed a standardized procedure. All ultrasound examinations were conducted by two experienced reproductive ultrasound diagnostic physicians. Before each examination, they all received unified training and strictly followed the measurement protocol. After measuring each sample three times, the average value was recorded. Measurement method: When measuring endometrial thickness, the uterine sagittal plane was selected to simultaneously display the endometrial ultrasound image from the cervical internal os to the uterine fundus. The maximum distance between the junction of the uterine muscle layer and the endometrium on both sides was taken. The unit is mm, and the measurement unit is accurate to 0.1 mm.

#### 2.2.4 Embryo status and pregnancy outcome criteria

MII ovum rate = MII ovum number/total retrieved ovum number × 100%; usable embryo rate = usable embryo number/2PN cleavage number × 100%; D3 high-quality embryo rate = D3 high-quality embryo number/2PN cleavage number × 100%; blastocyst formation rate = blastocyst number/blastocyst culture number × 100%; HCG positive: 2 weeks after transfer, check human chorionic gonadotropin (β-hCG), β-hCG >5 IU/L; HCG positive rate = the number of HCG-positive patients/the number of patients undergoing transfer cycle × 100%; clinical pregnancy: pregnancy sac (intrauterine or extrauterine) detected by vaginal ultrasound 4–5 weeks after transplantation; clinical pregnancy rate = number of clinical pregnancies/number of patients in transplantation cycles × 100%; abortion: embryos or fetuses were not viable and pregnancy was terminated; abortion rate = (number of early abortion cycles + number of late abortion cycles)/number of clinical pregnancy cycles; live births: live births; live birth rate = number of live birth cycles/number of transplant cycles (Table 2).

**Table 2:** Embryo status and pregnancy outcome criteria.

Indicator Name	Definition	Calculation Formula
MII ovum rate	The proportion of mature oocytes (MII stage) to the total number of retrieved ova	MII ovum rate = (MII ovum number/total retrieved ovum number) × 100%
Usable embryo rate	The proportion of usable embryos to the number of 2PN cleaved embryos	Usable embryo rate = (usable embryo number/2PN cleavage number) × 100%
D3 high-quality embryo rate	The proportion of high-quality embryos on Day 3 to the number of 2PN cleaved embryos	D3 high-quality embryo rate = (D3 high-quality embryo number/2PN cleavage number) × 100%
Blastocyst formation rate	The proportion of formed blastocysts to the number of embryos undergoing blastocyst culture	Blastocyst formation rate = (blastocyst number/blastocyst culture number) × 100%
HCG positive	Human chorionic gonadotropin (*β*-hCG) level >5 IU/L in peripheral blood 2 weeks after embryo transfer	HCG positive rate = (the number of HCG-positive patients/the number of patients undergoing transfer cycle) × 100%
Clinical pregnancy	Pregnancy sac (intrauterine or extrauterine) detected by transvaginal ultrasound 4–5 weeks after transplantation	Clinical pregnancy rate = (number of clinical pregnancies/number of patients in transplantation cycles) × 100%
Abortion	Termination of pregnancy when embryos or fetuses are not yet viable	Abortion rate = (number of early abortion cycles + number of late abortion cycles)/number of clinical pregnancy cycles × 100%
Live births	Delivery of viable fetuses	Live birth rate = (number of live birth cycles/number of transplant cycles) × 100%

### 2.3 Statistical analysis

SPSS 26.0 software was employed for data analysis. First, the one-sample nonparametric test was conducted on measurement data (Kolmogorov–Smirnov test) to determine whether it was normally distributed. Measurement data conforming to normal distribution are expressed as mean ± standard deviation ($\bar{x}\pm s$). Data with a non-normal distribution are expressed as median (P25, P75). Data with normal distribution were analyzed using ANOVA, and data with non-normal distribution were analyzed using the Kruskal–Wallis H test. Qualitative data are expressed as percentages. Such data were analyzed using the χ^2^-test. The correlations between each factor and the pregnancy outcome were analyzed using multivariate logistic regression, and the correlations between each factor and the embryo quality were analyzed using Beta regression. *p* < 0.05 was considered statistically significant.

## 3 Results

### 3.1 Comparison of general information, embryo quality, and pregnancy outcomes

**Figure 1:**
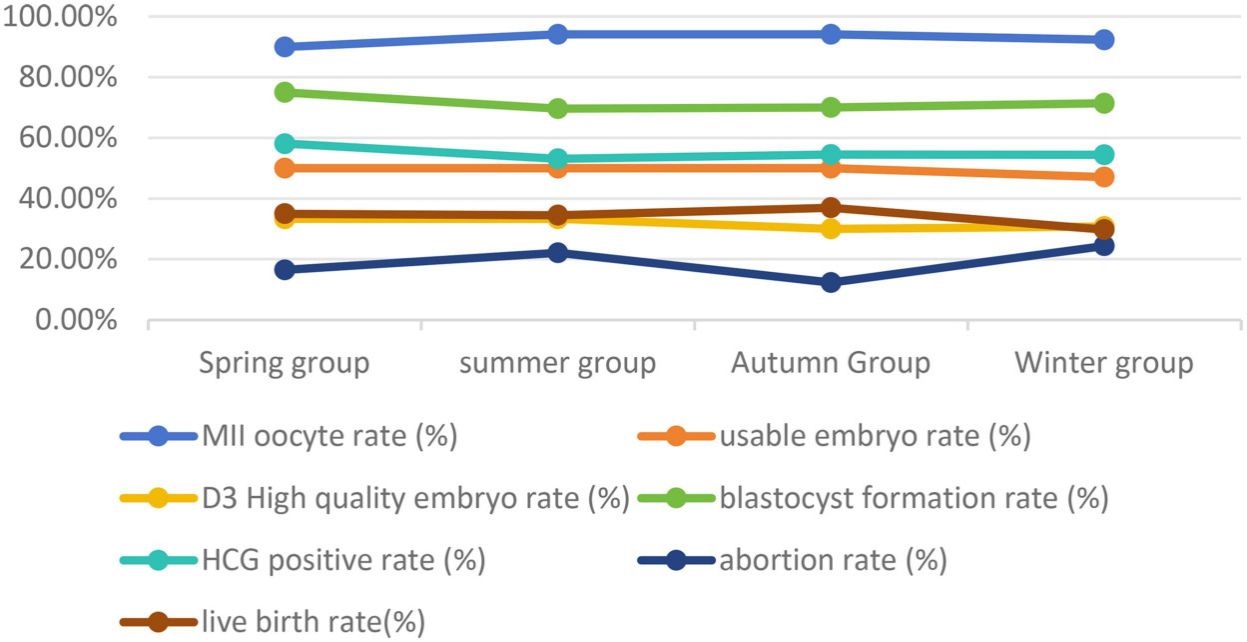
Line graphs showing the embryo quality and pregnancy outcomes among the four groups.

There were no significant differences among the four groups in terms of age, BMI, infertility type, total amount of Gn, basic FSH level, endometrial thickness on the day of transplantation, MII egg rate, usable embryo rate, D3 high-quality embryo rate, blastocyst formation rate, transplanting blastocysts, transplanting blastocysts, transplanting high-quality embryos, HCG positive rate, clinical pregnancy rate, abortion rate, and live birth rate (*P* > 0.05) (Table 3 and Figure 1).

**Table 3:** Comparison of general conditions, embryo quality and pregnancy outcomes of infertile patients.

Category		Spring group	summer group	Autumn Group	Winter group	P
Number of cycles (example)		431	423	305	254	
female age		31.00 (27.00–34.00)	31.00 (28.00–34.00)	31.00 (28.00–34.00)	31.00 (28.00–34.00)	0.852
BMI (Kg/m^2^)		22.60 (20.20–25.30)	22.30 (20.00–24.80)	22.27 (20.00–25.20)	22.03 (20.20–24.98)	0.650
Infertility type	Primary infertility	192 (44.5%)	190 (44.9%)	122 (40.0%)	104 (40.9%)	0.455
	Secondary infertility	239 (55.5%)	233 (55.1%)	183 (60.0%)	150 (59.1%)	
total amount of Gn		2,250.00 (1,613–2,775.00)	2,025.00 (1,413.00–2,700.00)	2,100.00 (1,500.00–2,700.00)	2,100.00 (1,500.00–2,700.00)	0.069
basic FSH（mIU/mL）		8.27 ± 2.08	8.25 ± 2.05	8.22 ± 2.25	8.19 ± 2.10	0.967
Endometrial thickness on day of transplantation		11.20 (9.70–13.40)	10.90 (9.50–12.40)	10.70,(9.20–12.60)	10.45 (9.30–12.35)	0.054
MII oocyte rate (%)		90.00% (80.00%–100.00%)	94.12% (80.00%–100.00%)	94.12% (81.82%–100.00%)	92.31% (81.82%–100.00%)	0.067
usable embryo rate (%)		50.00% (30.00%–66.67%)	50.00% (31.41%–66.67%)	50.00% (30.00%–66.67%)	47.06% (31.89%–63.80%)	0.696
D3 High quality embryo rate (%)		33.33% (16.67%–50.00%)	33.33% (16.67%–48.37%)	30.00% (14.64%–47.72%)	30.77% (17.40%–42.86%)	0.389
blastocyst formation rate (%)		75.00% (57.14%–85.71%)	69.62% (50.00%–83.30%)	70.00% (50.00%–83.33%)	71.40% (50.00%–83.33%)	0.056
Number of fresh embryo transfer cycles		203	194	154	114	
Blister blastocyst transfer		157 (77.3%)	168 (86.6%)	127 (82.5%)	94 (82.5%)	0.121
Blastocyst transfer		46 (22.7%)	26 (13.4%)	27 (17.5%)	20 (17.5%)	0.121
High-quality embryo transfer		177 (87.2%)	162 (83.5%)	132 (85.7%)	93 (81.6%)	0.537
HCG positive rate (%)		118 (58.1%)	103 (53.1%)	84 (54.5%)	62 (54.4%)	0.775
clinical pregnancy rate (%)		85 (41.9%)	86 (44.3%)	65 (42.2%)	45 (39.5%)	0.869
abortion rate (%)		14 (16.5%)	19 (22.1%)	8 (12.3%)	11 (24.4%)	0.299
live birth rate (%)		71 (35.0%)	67 (34.5%)	57 (37.0%)	34 (29.8%)	0.670

### 3.2The Impact of seasons on pregnancy outcomes

Multivariate binary logistic regression analysis was employed to account for the effect of confounding factors. The included factors were the age of women, BMI, type of the transplanted embryo (a binary variable, cleavage embryo = 1, blastocyst = 2), total Gn dose, season (with spring as the reference), and year (with 2019 as the reference). BMI was identified as an independent risk factor for HCG positive rate and clinical pregnancy rate (*P* < 0.05). There was a statistically significant association between the total Gn level and positive HCG (OR = 1.00, 95% CI: 0.999–1.000, *P* = 0.018). Season did not significantly affect HCG positive rate, clinical pregnancy rate, miscarriage rate, and live birth rate (Table 4).

**Table 4:** The impact of seasons on pregnancy outcomes.

Category	HCG positive		clinical pregnancy		abortion		live birth	
	OR（95%CI）	P	OR（95%CI）	P	OR（95%CI）	P	OR（95%CI）	P
female age	0.999 (0.960–1.039)	0.963	0.979 (0.941–1.018)	0.288	1.075 (0.990–1.167)	0.083	0.964 (0.925–1.005)	0.082
BMI (Kg/m2)	1.088 (1.039–1.140)	<0.001	1.064 (1.017–1.114)	0.007	1.038 (0.954–1.130)	0.382	1.045 (0.998–1.095)	0.063
Type of transplanted embryo	1.131 (0.745–1.719)	0.562	1.008 (0.665–1.526)	0.972	1.105 (0.488–2.500)	0.810	0.940 (0.610–1.450)	0.779
total amount of Gn season	1.000 (0.999–1.000)	0.018	1.000	0.436	1.000	0.829	1.000	0.417
Spring	1.000		1.000		1.000		1.000	
Summer	0.822 (0.545–1.240)	0.351	1.145 (0.760–1.725)	0.516	1.499 (0.670–3.350)	0.324	1.004 (0.657–1.536)	0.984
autumn	0.843 (0.547–1.298)	0.437	1.023 (0.665–1.576)	0.916	0.701 (0.269–1.825)	0.467	1.104 (0.709–1.719)	0.663
winter	0.844 (0.527–1.354)	0.482	0.921 (0.573–1.482)	0.734	1,672 (0.670–4.174)	0.271	0.798 (0.483–1.317)	0.377
year								
2019	1.000		1.000		1.000		1.000	
2020	0.761 (0.460–1.259)	0.761	0.860 (0.516–1.433)	0.563	2.488 (0.773–8.009)	0.127	0.704 (0.415–1.190)	0.195
2021	1.004 (0.590–1.709)	0.987	1.296 (0.762–2.203)	0.338	2.190 (0.679–7.063)	0.190	1.044 (0.606–1.798)	0.878
2022	0.925 (0.554–1.546)	0.767	0.896 (0.533–1.506)	0.679	2.091 (0.651–6.817)	0.221	0.762 (0.445–1.304)	0.321
2023	1.096 (0.644–1.866)	0.734	1.188 (0.700–2.017)	0.524	2.256 (0.693–7.347)	0.177	0.967 (0.561–1.668)	0.905

### 3.3 The impact of seasons on embryo quality

Beta regression analysis was used to account for the effect of confounding factors. The included factors were the age of women, BMI, total Gn dose, season (with spring as the reference), and year (with 2019 as the reference). The total Gn dose was negatively correlated with the blastocyst formation rate. The blastocyst formation rate in spring was significantly higher than that in summer. The MII oocyte rate in 2019 was significantly higher than that in 2020 and 2022. The available embryo rate in 2019 was significantly higher than that in 2022 and 2023 (*P* < 0.05) (Table 5).

**Table 5:** The impact of seasons on embryo quality.

Variables	Unstandardized coefficient		Standardization coefficient	*t*	*P*	Unstandardized coefficient		Standardization coefficient	*t*	*P*	Unstandardized coefficient		Standardization coefficient	*t*	*P*	Unstandardized coefficient		Standardization coefficient	*t*	*P*
	B	standard error (SE)	β			B	standard error（SE）	β			B	standard error（SE）	β			B	standard error（SE）	*Β*		
Intercept	97.338	16.752		5.811	<.001	81.143	5.017		16.173	<.001	62.216	6.504		9.566	<.001	43.100	5.907		7.297	<.001
female age	−0.590	0.331	−0.102	−1.785	0.075	0.205	0.122	0.048	1.679	0.093	−0.039	0.158	−0.007	−0.246	0.806	−0.160	0.144	−0.032	−1.115	0.265
BMI (Kg/m2)	−0.063	0.408	−0.009	−0.155	0.877	−0.064	0.142	−0.012	−0.452	0.652	−0.243	0.184	−0.035	−1.323	0.186	−0.132	0.167	−0.022	−0.788	0.431
total amount of Gn	−0.004	0.001	−0.116	−2.992	0.003	0.001	0.001	0.050	1.719	0.086	9.591	0.001	0.003	0.112	0.911	0.000	0.001	−0.010	−0.343	0.732
Season (Reference Group: Spring)																				
Summer	−9.220	3.564	−0.167	−2.587	0.010	2.226	1.244	0.056	1.789	0.074	1.180	1.613	0.022	0.731	0.465	0.116	1.465	0.003	0.079	0.937
autumn	−5.902	3.661	−0.104	−1.612	0.108	1.995	1.358	0.045	1.468	0.142	1.693	1.761	0.029	0.961	0.337	−1.214	1.599	-.024	-.759	0.448
winter	−2.126	4.187	−0.032	−0.508	0.612	1.189	1.439	0.025	0.826	0.409	−0.390	1.866	−0.006	−0.209	0.835	−1.752	1.694	-.032	−1.034	0.301
Year (Reference Group: 2019)																				
2020	0.497	4.795	0.008	0.104	0.918	−4.589	1.670	−0.105	−2.748	0.006	−4.020	2.165	−0.069	−1.857	0.064	−1.483	1.966	−0.029	−0.754	0.451
2021	8.197	4.858	0.136	1.687	0.093	−1.403	1.691	−0.031	−0.830	0.407	−2.513	2.192	−0.042	−1.146	0.252	0.415	1.991	0.008	0.209	0.835
2022	−1.638	4.675	−0.029	-.350	0.726	−4.757	1.654	−0.111	−2.876	0.004	−11.374	2.144	−0.199	−5.304	<.001	−1.459	1.947	−0.029	−0.749	0.454
2023	−3.907	4.925	−0.063	−0.793	0.428	−3.276	1.688	−0.073	−1.941	0.053	−17.052	2.189	−0.284	−7.791	<.001	−3.495	1.988	−0.066	−1.758	0.079
R2	0.068					0.020					0.068					0.007				
dependent variable	Blastocyst formation rate					MII oocyte rate					usable embryo rate					High-quality embryo transfer				

### 3.4 Hierarchical analysis based on years

The stratified analysis by year showed that in 2019, the total Gn volume in the spring group was significantly higher than that in the other groups. In 2020, the blastocyst formation rates in the spring and autumn groups were significantly higher than those in the winter group. In 2022, the HCG positive rate was significantly higher in the spring group than in the summer and autumn groups, and the clinical pregnancy rates were significantly higher in the spring and winter groups than in the summer group. In 2023, endometrial thickness on the day of transplantation was significantly higher in the spring group than in the winter group, and the blastocyst formation rate was significantly higher in the spring and winter groups than in the autumn group (*P* < 0.05) (Table 6).

**Table 6:** Stratified analysis by year.

Category		2019					2020	
		Spring group	summer group	Autumn Group	Winter group	P	Spring group	summer group
Number of cycles (example)		51	56	36	52		84	108
female age		31.94 ± 4.68	32.02 ± 4.54	31.47 ± 3.44	31.48 ± 4.40	0.884	29.80 ± 3.86	30.94 ± 4.08
		22.09 ± 3.57	22.76 ± 3.25	21.97 ± 3.02	22.39 ± 3.41	0.651	22.48 ± 3.50	22.17 ± 3.62
Infertility type	Primary infertility	17 (33.3%)	25 (44.6%)	11 (30.6%)	18 (34.6%)	0.494	38 (45.2%)	50 (46.3%)
	Secondary infertility	34 (66.7%)	31 (55.4%)	25 (69.4%)	34 (65.4%)		46 (54.8%)	58 (53.7%)
total amount of Gn		2,736.52 ± 715.84	2,385.94 ± 800.89	2,179.17 ± 583.98	2,181.25 ± 745.06	<0.001	2,202.88 ± 788.08	2,115.32 ± 728.20
Endometrial thickness on day of transplantation		11.30 ± 2.15	10.46 ± 2.32	10.10 ± 1.69	10.22 ± 1.81	0.11	10.41 ± 2.19	10.71 ± 2.72
Blister blastocyst transfer		25 (78.1%)	24 (82.8%)	19 (82.6%)	26 (100.0%)	0.106	30 (68.2%)	53 (86.9%)
Blastocyst transfer		7 (21.9%)	5 (17.2%)	4 (17.4%)	0 (0.0%)	0.106	14 (31.8%)	8 (13.1%)
High-quality embryo transfer		28 (87.5%)	28 (96.6%)	22 (95.7%)	21 (80.8%)	0.181	38 (86.4%)	51 (83.6%)
MII oocyte rate (%)		87.90 ± 17.16	91.69 ± 13.77	90.24 ± 14.59	91.00 ± 16.57	0.627	82.83 ± 22.68	87.18 ± 18.26
usable embryo rate (%)		50.40 ± 25.20	60.00 ± 23.89	56.97 ± 24.21	57.71 ± 24.79	0.223	51.99 ± 27.54	52.40 ± 25.33
D3 High quality embryo rate (%)		27.55 ± 19.07	39.35 ± 21.69	33.83 ± 22.18	33.84 ± 26.30	0.065	34.49 ± 23.26	32.94 ± 23.11
blastocyst formation rate (%)		63.77 ± 25.20	62.64 ± 21.97	70.15 ± 22.91	67.71 ± 21.98	0.658	70.54 ± 26.77	65.89 ± 23.39
HCG positive rate (%)		16 (50.0%)	17 (58.6%)	12 (52.2%)	15 (57.7%)	0.893	22 (50.0%)	33 (54.1%)
clinical pregnancy rate (%)		11 (34.4%)	14 (48.3%)	10 (43.5%)	9 (34.6%)	0.641	16 (36.4%)	28 (45.9%)
abortion rate (%)		1 (9.1%)	1 (7.1%)	2 (20.0%)	1 (11.1%)	0.79	4 (25.0%)	8 (28.6%)
live birth rate (%)		10 (31.3%)	13 (44.8%)	8 (34.8%)	8 (30.8%)	0.656	12 (27.3%)	20 (32.8%)

2020			2021					2022
Autumn Group	Winter group	P	Spring group	summer group	Autumn Group	Winter group	P	Spring group
80	40		92	90	56	51		117
30.04 ± 4.50	31.38 ± 4.15	0.284	31.30 ± 4.55	30.41 ± 4.21	30.81 ± 3.58	31.27 ± 3.90	0.424	30.77 ± 4.92
23.49 ± 3.44	22.78 ± 3.33	0.793	23.44 ± 3.52	23.16 ± 3.34	23.50 ± 3.81	23.35 ± 3.43	0.935	22.28 ± 3.57
37 (46.3%)	16 (40.0%)	>0.913	41 (44.6%)	41 (45.6%)	26 (46.4%)	26 (51.0%)	>0.901	57 (48.7%)
43 (53.8%)	24 (60.0%)		51 (55.4%)	49 (54.4%)	30 (53.6%)	25 (49.0%)		60 (51.3%)
2,109.70 ± 901.09	2,379.55 ± 645.74	0.579	2,345.55 ± 816.72	2,250.87 ± 837.9	2,124.13 ± 888.81	2,440.97 ± 862.49	0.318	2,104.55 ± 717.95
11.45 ± 2.40	11.75 ± 2.67	0.126	12.13 ± 2.53	11.61 ± 2.36	10.58 ± 1.99	11.44 ± 2.41	0.088	11.14 ± 2.54
30 (75.0%)	12 (70.6%)	0.121	29 (72.5%)	32 (86.5%)	20 (80.0%)	12 (57.1%)	0.081	38 (90.5%)
10 (25.0%)	5 (29.4%)	0.121	11 (27.5%)	5 (13.5%)	5 (20.0%)	9 (42.9%)	0.081	4 (9.5%)
33 (82.5%)	15 (83.3%)	0.967	35 (87.5%)	30 (81.1%)	23 (92.0%)	19 (90.5%)	0.587	38 (90.5%)
83.62 ± 22.82	88.71 ± 18.91	0.346	88.02 ± 16.04	89.85 ± 18.19	88.95 ± 12.76	86.28 ± 14.55	0.665	85.01 ± 19.74
53.07 ± 24.42	51.34 ± 23.63	0.975	54.60 ± 24.04	53.69 ± 22.39	55.44 ± 23.36	50.08 ± 19.76	0.62	46.69 ± 23.19
31.49 ± 21.06	30.84 ± 21.75	0.775	34.74 ± 19.77	33.26 ± 19.28	36.47 ± 22.33	35.4 ± 16.18	0.786	36.08 ± 20.74
74.4 ± 22.51	52.92 ± 30.71	0.022	76.44 ± 23.62	74.68 ± 20.51	70.06 ± 28.04	74.79 ± 26.30	0.68	72.19 ± 21.71
19 (47.5%)	7 (38.9%)	0.707	22 (55.0%)	22 (59.5%)	14 (56.0%)	13 (61.9%)	0.921	30 (71.4%)
16 (40.0%)	3 (16.7%)	0.161	18 (45.0%)	20 (54.1%)	11 (44.0%)	11 (52.4%)	0.807	21 (50.0%)
1 (6.3%)	0 (0.0%)	0.255	2 (11.1%)	5 (25.0%)	2 (18.2%)	3 (27.3%)	0.662	4 (19.0%)
15 (37.5%)	3 (16.7%)	0.407	16 (40.0%)	15 (40.5%)	9 (36.0%)	8 (38.1%)	0.984	17 (40.5%)
2022年				2023				
summer group	Autumn Group	Winter group	P	Spring group	summer group	Autumn Group	Winter group	P
94	64	55		87	75	69	56	
30.50 ± 3.85	31.25 ± 44.59	30.62 ± 4.00	0.735	30.09 ± 4.09	31.27 ± 4.17	31.65 ± 4.06	30.82 ± 4.14	0.479
22.55 ± 3.43	23.24 ± 3.65	23.17 ± 3.75	0.658	23.73 ± 3.32	22.92 ± 3.39	22.75 ± 3.33	22.13 ± 3.69	0.049
44 (46.8%)	22 (34.4%)	19 (34.5%)	>0.129	39 (44.8%)	30 (40.0%)	26 (37.7%)	25 (44.6%)	>0.778
50 (53.2%)	42 (65.6%)	36 (65.5%)		48 (55.2%)	45 (60.0%)	43 (62.3%)	31 (55.4%)	
1,963.31 ± 840.39	2,235.97 ± 826.53	2,049.48 ± 816.10	0.084	2,211.78 ± 871.38	2,182.33 ± 897.72	2,163.23 ± 733.95	2,149.48 ± 809.42	0.963
11.96 ± 2.33	10.78 ± 2.78	9.92 ± 2.14	0.11	12.78 ± 3.10	11.26 ± 2.63	11.77 ± 2.47	11.00 ± 2.03	0.037
37 (90.2%)	30 (85.7%)	24 (88.9%)	0.909	35 (77.8%)	22 (84.6%)	28 (90.3%)	19 (86.4%)	0.572
4 (9.8%)	5 (14.3%)	3 (11.1%)	0.909	10 (22.2%)	4 (15.4%)	3 (9.7%)	3 (13.6%)	0.572
33 (80.5%)	27 (77.1%)	22 (81.5%)	0.439	38 (84.4%)	20 (76.9%)	27 (87.1%)	16 (72.7%)	0.497
83.73 ± 19.73	86.02 ± 16.18	84.71 ± 20.69	0.905	84.29 ± 15.81	87.24 ± 17.81	84.98 ± 18.27	85.10 ± 17.12	0.219
43.24 ± 22.57	45.77 ± 23.31	42.78 ± 22.69	0.619	36.55 ± 20.64	40.88 ± 21.17	40.52 ± 24.42	39.50 ± 19.20	0.562
33.01 ± 21.13	27.99 ± 20.70	30.14 ± 18.34	0.06	29.81 ± 20.18	30.80 ± 22.55	31.42 ± 23.48	29.57 ± 18.92	0.954
62.33 ± 18.24	65.05 ± 20.18	66.32 ± 22.66	0.122	66.03 ± 23.79	59.72 ± 27.45	48.47 ± 26.79	69.38 ± 23.73	0.005
17 (41.5%)	17 (48.6%)	17 (63.0%)	0.031	28 (62.2%)	14 (53.8%)	22 (71.0%)	10 (45.5%)	0.264
10 (24.4%)	12 (34.3%)	14 (51.9%)	0.046	19 (42.2%)	14 (53.8%)	16 (51.6%)	8 (36.4%)	0.498
3 (30.0%)	0 (0.0%)	4 (28.6%)	0.223	3 (15.8%)	2 (14.3%)	3 (18.8%)	3 (37.5%)	0.556
7 (17.1%)	12 (34.3%)	10 (37.0%)	0.111	16 (35.6%)	12 (46.2%)	13 (41.9%)	5 (22.7%)	0.359

## 4 Discussion

Natural environment, residence, and lifestyle are important factors affecting reproductive health. High environmental temperatures are associated with reduced ovarian reserve and reproductive aging in women (19). Studies have shown that all organisms will experience heat stress when the environmental temperature exceeds 25 °C (20–22). Heat stress changes the maternal reproductive system. Granulosa cells in the follicles affect the development of oocytes by producing steroids (23). Heat stress significantly lowers the survival rate of granulosa cells, inhibits steroid production, limits the proliferation and transformation of granulosa cells, intensifies oxidative stress, and enhances the apoptosis of granulosa cells. Heat stress can alter the follicular microenvironment, such as the concentration of amino acids, fatty acids, minerals, enzymes, antioxidants, and growth factors, in follicular fluid, resulting in abnormal oocyte development ability and quality (24). This study found that the blastocyst formation rate was higher in the spring group than in the summer group. Although the difference was not statistically significant (*P* = 0.056), after adjusting for confounding factors through Beta regression analysis, the blastocyst formation rate in the spring group was significantly higher than that in the summer group (*P* < 0.05). The average temperature in summer was higher than 25 °C, and heat stress reduced the developmental ability of oocytes, resulting in a lower blastocyst formation rate in summer. This study also found that the formation rate of blastocysts in autumn and winter was higher than that in summer (*P* > 0.05). Even after adjusting for confounding factors through Beta regression analysis, the result still did not reach a statistically significant level. It is possible that the high temperature in summer is persistent, while the temperature in autumn (especially early autumn) gradually decreases, but there may still be short-term periods of high temperature; in winter, indoor heating may weaken the protective effect of low temperature, resulting in insufficient gradient of temperature difference between autumn-winter and summer; and the shortening of daylight hours and increased risk of vitamin D deficiency in autumn-winter may counteract the potential benefits of low temperature on blastocyst formation, leading to no statistically significant difference between autumn-winter and summer.

Currently, there are relatively few studies on the relationship between seasonal changes and the outcome of IVF-ET-assisted pregnancy. Some studies have reported that different seasons affect the outcome of IVF-ET-assisted pregnancy, while others claim that different seasons do not affect the outcome. This study found no significant differences among the four groups (spring, summer, autumn, and winter groups) in terms of female age, BMI, infertility type, total amount of Gn, basic FSH level, endometrial thickness on day of transplantation, MII ovum rate, usable embryo rate, D3 high quality embryo rate, transplanting blastocysts, transplanting blastocysts, transplanting high-quality embryos, HCG positive rate, clinical pregnancy rate, abortion rate, and live birth rate (*P* > 0.05). After adjusting for confounding factors using binary logistic regression analysis, season did not significantly affect the positive rate of HCG, clinical pregnancy rate, miscarriage rate, and live birth rate. After using Beta regression analysis to adjust for confounding factors, season did not significantly affect the MII oocyte rate, available embryo rate, and D3 high-quality embryo rate. In *in vitro* fertilization, sperm and eggs complete the fertilization process in an artificially controlled environment. Human-assisted reproduction (ART) laboratory is an important place for *in vitro* fertilization and embryo culture, and a major component of human-assisted reproduction center (25). Human-assisted reproduction laboratory has strict requirements. Embryo laboratory generally necessitates 1000-class purification, operation area necessitates 100-class purification, temperature is fixed at 22 ℃–25 ℃, relative humidity is controlled at 40%–60%, minimum illumination is ≥350 LX, and noise is ≤50 dB. Air cleanliness requires air bacterial culture <0.2 cfu/30 min in a 1,000-class area, air bacterial culture <0.4 cfu/30 min in a 1,000-class area and <¢90 mm plate. It is important to keep the air clean, keep the temperature, humidity, and pressure constant, and control noise as much as possible to prevent the contamination of the incubator by bacteria and dust (26). The constant environment in the laboratory minimizes the effect of external temperature on the quality of embryos. Multiple factors, such as embryo quality, endometrial receptivity, and endocrine conditions, affect the outcome of IVF-ET pregnancy. Correia et al. (27) indicated that seasonal or environmental conditions affect the development and maturation of oocytes, but do not affect endometrial receptivity and early pregnancy development. The human body is categorized as a warm-blooded animal. In cases of changes in the ambient temperature, the human body temperature can adjust through the peripheral temperature sensor and the central temperature sensor. The heat production and heat dissipation process maintains a dynamic balance to adapt to the changes in ambient temperature and keep the human body temperature constant at around 37 ℃. This ensures the stability of the internal environment, the normal operation of various enzymatic reactions, cell metabolism, and physiological functions (28). Although there are significant differences in seasons and temperatures, the internal environment can stabilize through the body's autoregulation ability, thereby ensuring a stable outcome of IVF-ET pregnancies.

After stratification by year, in 2019, the total amount of Gn in the spring group was significantly higher than that in the other groups. Compared to the winter group, the blastocyst formation rates were significantly higher in the spring and autumn groups in 2020 and in the autumn group in 2022. In 2022, the HCG positive rate in the spring group was significantly higher than that in the summer and autumn groups, and the clinical pregnancy rates in the spring and winter groups were significantly higher than those in the summer group (*P* < 0.05). In the overall analysis, season did not significantly affect the outcomes of pregnancy, possibly because the seasonal effects in different years attenuate each other's effect. After stratification by year, significant advantages were observed in 2020 spring and autumn, 2022 spring and winter, and 2023 spring and winter. There may have been potential interactions between years and seasons. Our research also found that, when analyzed as a whole, the endometrial thickness on the transplantation day in spring was higher than that in summer, autumn, and winter. Although the difference was not statistically significant (*P* = 0.056), after stratification by year, the endometrial thickness on the transplantation day in the spring group in 2023 was significantly higher than that in the winter group (*P* = 0.037). The suitable environmental temperature in spring may regulate the secretion rhythm of estrogen and progesterone in the body, improve the blood perfusion of the endometrium, promote the proliferation of endometrial stromal cells and angiogenesis, thereby increasing the endometrial thickness; while the low-temperature environment in winter may inhibit the function of the reproductive endocrine axis and slow down the growth rate of the endometrium. The specific occurrence of this difference in 2023 might be related to the stable temperature rise in spring and no significant interference from cold waves that year. Stable climatic conditions are more conducive to the benign development of the endometrium.

In summary, the results of this study showed that: The formation rate of blastocysts in spring was higher than that in summer, and there may have been potential interactions between the year and the season. This study accounted for the effects of systemic diseases, hydrosalpinx, uterine diseases, and genetic factors on the results through strict inclusion and exclusion criteria. In addition, to avoid the effect of different controlled ovarian stimulation protocols on the outcome, this study only included patients who used antagonist regimens for controlled ovarian stimulation protocols. The effect of confounding factors was minimized by taking these measures. This study was a retrospective single-center study, and a prospective randomized controlled study can be conducted to avoid the effect of different regions on the outcome. It is also possible to comprehensively explore how different seasons affect the IVF-ET-assisted pregnancy outcomes by year.

## Data Availability

The original contributions presented in the study are included in the article/Supplementary Material, further inquiries can be directed to the corresponding author.
